# Effect of transforaminal epidural polydeoxyribonucleotide injections on lumbosacral radiculopathy: A case report

**DOI:** 10.1097/MD.0000000000007174

**Published:** 2017-06-23

**Authors:** Keum Nae Kang, Tae Woong Kim, Jin Woo Koh, Han Byeol Oh, Jong-Uk Mun, Mi Sook Seo, Young Uk Kim

**Affiliations:** a Department of Anesthesiology and Pain Medicine, National Police Hospital, Seoul; b Department of Orthopaedic Surgery, Changwon Gyeongsang National University Hospital; c Department of Anesthesiology and Pain Medicine, Catholic Kwandong University of Korea College of Medicine, International St. Mary's Hospital, Incheon, Republic of Korea.

**Keywords:** case report, diabetes mellitus, glucocorticoids, lumbosacral radiculopathy, polydeoxyribonucleotide, transforaminal epidural block

## Abstract

**Rationale:**

Transforaminal epidural glucocorticoids administration is widely performed for the management of lumbosacral radiculopathy. However, it may worsen the condition of patients with type 2 diabetes mellitus (DM). Polydeoxyribonucleotide (PDRN) was recently noted as a substitute for glucocorticoids.

**Patient concerns:**

A 44-year-old male patient was admitted to our pain clinic with symptoms of low back pain with severe pain and tingling sensation of left posterolateral leg. He had type 2 DM medicated with Glimepiride and Metformin. Blood glucose level was 367 mg/dL. He declined to use glucocorticoid.

**Diagnoses:**

He was diagnosed as left foraminal disc protrusion at L4–5, left subarticular disc protrusion at L5-S1.

**Interventions:**

Fluoroscopically guided transforaminal epidural PDRN injections were carried out.

**Outcomes:**

The patient was followed up for more than 6 months and demonstrated good improvement in lumbosacral radiculopathy without any complications.

**Lessons:**

This is the first successful report on epidural injection of PDRN.

## 1 Introduction

Administration of transforaminal epidural glucocorticoids is common for the management of lumbosacral radiculopathy and low back pain originating from lumbar intervertebral disc herniation.^[1–4]^ Although injection of synthetic glucocorticoids is beneficial due to their antiinflammatory effects,^[5]^ the use of glucocorticoids is limited as it may cause multiple adverse effects such as glucocorticoid-induced osteoporosis,^[6]^ adrenal suppression,^[7]^ and cognitive and mood disorders.^[7]^ Glucose intolerance associated with decreased insulin sensitivity is a major concerns related to use of synthetic glucocorticoids, and it may worsen the condition of patients with type 2 diabetes mellitus (DM).^[8]^

Many studies have sought substitutes for glucocorticoids. Polydeoxyribonucleotide (PDRN, Placentexingergro; Mastellisrl, San Remo, Italy) was recently noted as such a substitute.^[9]^ PDRN has antiinflammatory effects, as it lowers the expression of inflammatory cytokines including interleukin-6 and tumor necrosis factor-alpha. PDRN has not displayed any adverse effects.^[9]^

This case report concerns a patient with type 2 DM who underwent transforaminal epidural PDRN injection for the management of lumbosacral radiculopathy.

## 2 Case presentation

A 44-year-old male patient with type 2 DM was admitted to the emergency room with symptoms of low back pain with severe pain and tingling sensation of left posterolateral thigh, knee and posterior part of lower leg, numbness of left foot dorsum and big toe, and paresthesia in the left posterolateral thigh. The pain was exacerbated when he bent down. The patient reported baseline numbness that began after playing soccer 30 days before. At the time of admittance, the patient was bedridden and could not walk due to severe pain, which he rated 8/10 on a numeric rating scale (NRS). Neurological examination revealed no weakness and reflex abnormality. The straight leg raising test and femoral nerve stretching test were carried out. Straight leg raising test was positive in the left leg. Femoral nerve stretching test was negative. His height was 178.6 cm and his weight was 80.6 kg, with a body mass index of 25.27 kg/m^2^. Diabetes medications included Glimepiride and Metformin; blood glucose level was 367 mg/dL.

**Figure 1:**
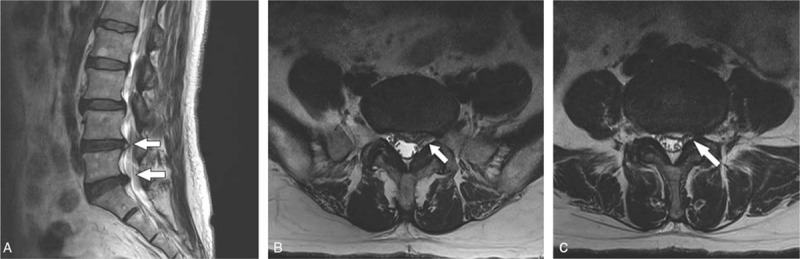
Sagittal T2-weighted magnetic resonance imaging (MRI) showing L4–5 and L5–S1 disc herniation (A). Axial view of MRI shows left foraminal disc protrusion at L4–5 (B), left subarticular disc protrusion at L5–S1 (C).

Lumbar magnetic resonance imaging revealed left foraminal disc protrusion at L4–5, left subarticular disc protrusion at L5–S1 (Fig. 1). The patient was advised of glucocorticoid-related side effects, such as glucose intolerance associated with decrease in insulin sensitivity. He declined to use glucocorticoid. When the antiinflammatory effect of PDRN was explained to the patient, he provided written informed consent for its use.

**Figure 2:**
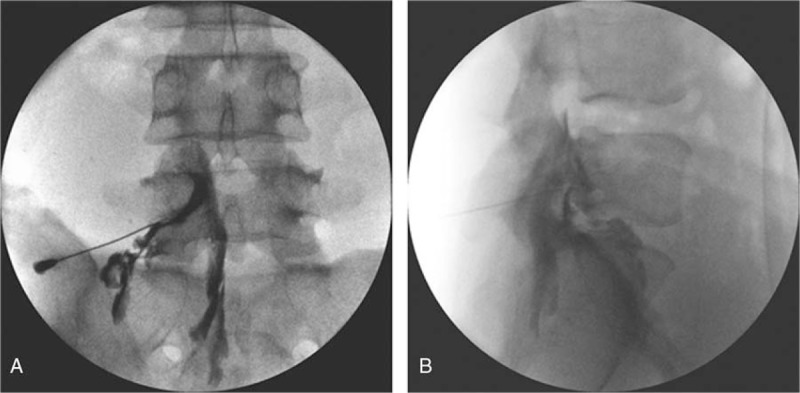
An anteroposterior live fluoroscopic image taken during a contrast injection for a left L5 transforaminal epidural steroid injection (A). Lumbar lateral view revealing a contrast medium in the anterior epidural space (B).

The patient was brought to the fluoroscopy room and placed prone on the table. Fluoroscopically guided transforaminal epidural block was performed with PDRN at the level of the L4, L5 spinal nerve roots using 5.625 mg/3 mL of PDRN, 0.3% lidocaine 5 mL with a 22-G, 9-cm needle (Fig. 2). At the 1 week follow-up after the first transforaminal epidural PDRN injection, the NRS score had decreased from 8 to 5, but the patient reported that his left leg was still painful. No adverse-reactions were observed. Therefore, transforaminal epidural PDRN injection mixed with 0.3% lidocaine was delivered into the same site. In the follow-up treatments, the PDRN injection was repeated 3 times at 1 week intervals under fluoroscopic guidance. The patient reported significant pain reduction with decreased NRS scores from 5 to 1. The patient was followed up for more than 6 months and demonstrated good improvement in pain without any complications.

## 3 Discussion

A male patient with type 2 DM with barely controlled glucose level successfully received transforaminal epidural PDRN injections for lumbosacral radiculopathy. When glucose level is being barely controlled, the use of glucocorticoids has a high risk of acute or chronic complications associated with DM. Glucocorticoids destabilize glucose homeostasis and impair glucose tolerance in patients receiving steroid therapy.^[8]^ This unwanted action of glucocorticoids is related to the suppression of the hypothalamic-pituitary-adrenal axis.^[10]^ Ward et al reported the case of a 65-year-old man with type 2 DM who experienced hyperosmolar nonketotic hyperglycemic coma 24 hours after the epidural administration of triamcinolone 80 mg for low back pain and sciatica. The authors reported that caudal epidural injection with triamcinolone resulted in significant insulin sensitivity change in patients whose glucose tolerance was normal.^[11]^ Furthermore, glucocorticoids use increases the risk of osteoporosis which is concerned with bone apoptosis, bone resorption, calcium balance, osteoblast function, and sex hormone.^[12]^ Multiple endocrine and metabolic adverse effects of glucocorticoids, such as glucocorticoid-induced osteoporosis, steroid-related adrenal insufficiency, and cognitive and mood disorders,^[7]^ brought about the need of new agents to substitute for glucocorticoids.

Clonidine, disease modifying antirheumatic drugs (DMARDs), and PDRN have been spotlighted as alternatives to glucocorticoids for epidural injection. Injection of clonidine, an alpha-2 agonist, into the epidural space has an effect on radiculopathy^[13]^ due to its antiinflammatory and analgesic effects. However, clonidine also has unwanted effects that include increased potassium conductivity, blockage of C and A-δ fibers, and local anesthetic blockade enhancement.^[14]^ Epidural space injections of etanercept and tocilizumab as DMARDs have been investigated. Cohen et al^[15]^ studied the efficacy of transforaminal epidural etanercept injection; etanercept improved the patient's symptoms compared to placebo. Ohtori et al^[16]^ demonstrated that transforaminal epidural tocilizumab injection was more effective than placebo in relieving pain and numbness in lower extremities and back. In spite of the efficacy of DMARDS, we had difficulty in applying these agents to the patient, due to concerns about drug toxicity and infection from immunosuppression.

On the other hand, PDRN, whose action is derived from antiinflammatory effects that promote wound healing by tissue regeneration, is known to be safe. PDRN has been widely used in many fields recently; however, its drug-related toxicity has not been reported.^[9]^ PDRN is a nucleoside that repairs and regenerates cellular damage by interacting with A2 purinergic receptor and accelerating the differentiation of fibroblasts.^[17]^ Kim et al^[18]^ established that PDRN injection is effective in patients with ischiofemoral impingement syndrome who are not indicated for surgery. Lim et al^[19]^ reported the effectiveness of PDRN injection in posterior tibial tendon dysfunction patients undergoing ankle syndesmotic surgery. The indications of the safety and efficacy of PDRN prompted its use in the present case. Three transforaminal epidural PDRN injections obtained satisfactory results without any unwanted effects.

This is the first report on the epidural injection of PDRN, although a number of studies have been made on other routes of PDRN administration. We suggest that further studies on the efficacy and safety of PDRN are necessary, dealing with the diverse use of PDRN.
